# Acute heat stress activated inflammatory signaling in porcine oxidative skeletal muscle

**DOI:** 10.14814/phy2.13397

**Published:** 2017-08-22

**Authors:** Shanthi Ganesan, Olga Volodina, Sarah C. Pearce, Nicholas K. Gabler, Lance H. Baumgard, Robert P. Rhoads, Joshua T. Selsby

**Affiliations:** ^1^ Department of Animal Science Iowa State University Ames Iowa; ^2^ Department of Animal and Poultry Sciences Virginia Tech Blacksburg Virginia

**Keywords:** AP‐1, heat stroke, hyperthermia, inflammation, Jak Stat, NF‐*κ*B

## Abstract

Despite well‐studied clinical manifestations, intracellular mechanisms of prolonged hyperthermic injury remain unclear, especially in skeletal muscle. Given muscle's large potential to impact systemic inflammation and metabolism, the response of muscle cells to heat‐mediated injury warrants further investigation. We have previously reported increased activation of NF‐*κ*B signaling and increased NF‐*κ*B and AP‐1‐driven transcripts in oxidative skeletal muscle following 12 h of heat stress. The purpose of this investigation was to examine early heat stress‐induced inflammatory signaling in skeletal muscle. We hypothesized that heat stress would increase NF‐*κ*B and AP‐1 signaling in oxidative skeletal muscle. To address this hypothesis, 32 gilts were randomly assigned to one of four treatment groups (*n* = 8/group): control (0 h: 21°C) or exposed to heat stress conditions (37°C) for 2 h (*n* = 8), 4 h (*n* = 8), or 6 h (*n* = 8). Immediately following environmental exposure pigs were euthanized and the red portion of the semitendinosus muscle (STR) was harvested. We found evidence of NF‐*κ*B pathway activation as indicated by increased protein abundance of NF‐*κ*B activator IKK‐*α* following 4 h and increased total NF‐*κ*B protein abundance following 6 h of heat stress. Heat stress also stimulated AP‐1 signaling as AP‐1 protein abundance was increased in nuclear fractions following 4 h of heat stress. Interleukin‐6 protein abundance and activation of the JAK/STAT pathway were decreased in heat stressed muscle. These data indicate that heat stress activated inflammatory signaling in the porcine STR muscle via the AP‐1 pathway and early activation of the NF‐*κ*B pathway.

## Introduction

Chronic and acute heat‐related illnesses, including heat stroke, remain a major consequence of global warming with the likelihood of increased incidence given current predictive environmental models. Prolonged environmental hyperthermia can result in clinical manifestations that can vary from exacerbation of cardiovascular risk factors such as hypertension (Fonseca et al. 2015) or disruption of the intestinal barrier (Xu et al. 2015) to potentially life‐threatening diseases, including kidney pathology (Glaser et al. 2016), coronary artery disease, or cardiac arrest (Kones 2011). This chronic hyperthermic stress can affect not only human health, but also agricultural animal welfare and decrease agricultural productivity and growth efficiency implicating hyperthermic muscle injury in this process. For example, the U.S. swine industry is conservatively estimated to lose more than $900 million annually, mostly from decreased meat production (Baumgard and Rhoads 2013) and reduced fertility (Nteeba et al. 2015). While the negative phenotypic effects of heat stress are clearly established, little is known about molecular changes that underlie heat‐induced injury. The limited mechanistic understanding of hyperthermic injury contributes to the absence of etiological and globally agreed upon interventions for heat illnesses and strategies to ameliorate heat stress‐mediated losses in agricultural production. Given the genotypic and phenotypic similarities between pigs and humans (Humphray et al. 2007), it is likely that the two mammals share heat‐induced pathologies and thus studying hyperthermic injury in a pig model will dually benefit human health and agricultural production.

Prolonged hyperthermic exposure appears to be in stark contrast to therapeutic hyperthermia during which brief (approximately 30 min) hyperthermia attenuates muscle atrophy (Naito et al. 2000; Selsby and Dodd 2005), augments muscle growth (Selsby et al. 2007; Takeuchi et al. 2014) and maintains insulin sensitivity (Gupte et al. 2011). Prolonged hyperthermia also appears distinct from changes occurring in models of heat stroke, which are often approximately 2 h in duration and may include a component of exercise. In particular, interleukin‐6 (IL‐6) is strongly induced in muscle during heat stroke and appears to be cytoprotective for a number of organ systems (Welc et al. 2012), however, it is blunted during 12 h of hyperthermic exposure (Ganesan et al. 2016). Moreover, heat stroke appears to induce inflammatory signaling via AP‐1 but not NF‐*κ*B (Welc et al. 2013b), while more prolonged hyperthermic exposure activates both AP‐1 and NF‐*κ*B (Ganesan et al. 2016).

In skeletal muscle, we have previously established that 12 h of environmental hyperthermia induced NF‐*κ*B signaling, which returned to baseline following 24 h (Montilla et al. 2014). Of interest, it was clear that NF‐*κ*B signaling was activated in muscle prior to the 12 h time point as indicated by increased expression of NF‐*κ*B‐driven genes (Ganesan et al. 2016). Abundance of AP‐1‐driven transcripts was also increased though AP‐1 signaling was not evident. This raised the possibility of a complex chronology of inflammatory signaling in skeletal muscle occurring earlier in the response to environmental hyperthermia. LPS can activate inflammatory signaling (Song et al. 2016) and endotoxemia appears to be a consistent effect of heat stress (Pearce et al. 2012, 2013a,c). Moreover, we found recently that 12 h of heat stress led to oxidative stress (Ganesan et al. 2017) and that it has long been appreciated that oxidative stress can promote inflammatory signaling (Ambade and Mandrekar 2012). Importantly, we have recently demonstrated increased oxidative stress in these muscles (Volodina et al. 2017). Given the possibility of inflammatory signaling driven by LPS and/or oxidative stress, the purpose of this investigation was to determine the extent to which inflammatory signaling was increased following short‐term heat stress in skeletal muscle. We hypothesized that 2, 4, and 6 h of environmental hyperthermia would result in activated inflammatory signaling via NF‐*κ*B and AP‐1 pathways in oxidative skeletal muscle.

## Materials and Methods

### Animals and study design

All procedures were reviewed and approved by the Iowa State University Institutional Animal Care and Use Committee (protocol #2‐12‐7307‐S). A detailed experimental design and phenotypic data have been previously published (Pearce et al. 2014). Briefly, 32 gilts with average body weight of 63.8 ± 2.9 kg were randomly assigned to four groups. Control pigs (*n* = 8) were kept at thermoneutral conditions for 6 h (0 h heat stress; 21°C; ~70% humidity), while animals from other groups were exposed to heat stress conditions (37°C; ~40% humidity) for 2 h (*n* = 8), 4 h (*n* = 8), or 6 h (*n* = 8). Ad libitum access to feed and water was provided for all animals, and the diet met the requirements of the Subcommittee on Swine Nutrition, Committee on Animal Nutrition, Board on Agriculture, and National Research Council (1998) for swine feed and management. Environmental temperature and humidity were recorded every 5 min by a data recorder (Lascar^®^ EL‐USB‐2‐LCD, Erie, PA). Rectal temperature, respiratory rate, and feed intake were recorded every two hours. Once the environmental treatment was finished, animals were killed by barbiturate overdose and exsanguination and semitendinosus red muscle (STR) was harvested immediately and stored at −80°C. STR was selected for analysis as we have previously discovered a high degree of hyperthermic dysfunction while the glycolytic, white portion of the ST is comparatively resistant to hyperthermia.

### Protein extraction and immunochemistry

Procedures were performed as recently described (Ganesan et al. 2016). Protein was extracted according to standard techniques. The nuclear fraction was isolated using a Nuclear Extraction Reagents Kit according to manufacturer instructions (Thermo Fischer Scientific, Inc., Waltham, MA). Protein concentration was normalized following a BCA assay (Thermo Fischer Scientific, Inc., Waltham, MA), and protein was diluted in Laemmli buffer to 4 mg/mL and boiled for 5 min to denature proteins. Forty micrograms of protein were separated on PAGEr Gold Precast Gels (Lonza, Walkersville, MD) at 120 V and transferred to 0.2 *μ*m pore‐size nitrocellulose membranes (Bio‐Rad, Hercules, CA) at 100 V for 1 h at 4°C. Equal loading was verified by quantification of Ponceau‐S staining and all membranes were similar between groups. Membranes were exposed to primary antibodies overnight at 4°C (Table 1). After washing, membranes were exposed to appropriate anti‐mouse or anti‐rabbit secondary antibody (Cell Signaling Technology) for 1 h at room temperature, washed and incubated with ECL Western Blotting Substrate (Thermo Fischer Scientific, Inc., Waltham, MA) for approximately 5 min at room temperature. Blots were imaged on X‐ray film (Phenix Research Products, Candler, NC), and the resultant bands quantified with Carestream software, using the automated band quantification feature where possible to limit bias. As an additional control, membranes were exposed to secondary antibody only and no signal was obtained.

**Table 1:** Antibodies and dilutions used in immunoblotting

Antibodies	Primary dilution	Secondary dilution	Company/Product no
Heat shock protein (HSP70)	1:1000	1:2000	Enzo Life Sciences: C95F3A‐5
Heat shock protein (HSP60)	1:750	1:2000	Cell signaling Technology: 12165
Heat shock protein (HSP90)	1:1000	1:3000	Cell signaling Technology: 4874
Stress‐activated protein kinases (SAPK)/c‐Jun N‐terminal kinases (JNK)	1:1000	1:3000	Cell signaling Technology: 9251
phospho‐SAPK/JNKThr183/Tyr185	1:1000	1:2000	Cell signaling Technology: 9251
Nuclear factor kappa‐light‐chain‐enhancer of activated B cells (NF‐*κ*B p65)	1:5000	1:3000	Cell signaling Technology: 8242
phospho‐NF‐*κ*B p65^Ser536^	1:1000	1:1000	Cell signaling Technology: 3033
Nuclear factor kappa‐alpha kinase subunit alpha (IKK*α*)	1:1000	1:2000	Cell signaling Technology: 2682
Nnuclear factor of kappa light polypeptide gene enhancer in B‐cells inhibitor, alpha (IKB*α*)	1:2000	1:2000	Cell signaling Technology: 9242
Tumor necrosis factor alpha (TNF*α*)	1:2000	1:2000	Cell signaling Technology: 3707
Interleukin‐6 (IL‐6)	1:500	1:2000	Cell signaling Technology: 12153
Interleukin‐1*β*	1:500	1:2000	Cell signaling Technology: 12242
Janus Kinase (JAK1)	1:1000	1:2000	Cell signaling Technology: 3341
Janus Kinase (JAK2)	1:1000	1:2000	Santa Cruz Biotechnology: SC294
Signal transducer and Activator of Transcription (STAT3)	1:1000	1:3000	Cell signaling Technology: 4904
phospho‐STAT3^Tyr705^	1:750	1:1000	Cell signaling Technology: 9131

### mRNA isolation and RT‐qPCR

mRNA was isolated as recently described (Ganesan et al. 2016). Briefly, mRNA was isolated from 50 mg of powdered muscle homogenized in Trizol (Invitrogen, Carlsbad, CA), applied to a Direct‐zol MiniPrep column (Zymo, Irvine, CA), and treated with DNase. mRNA concentration was measured, using an ND‐1000 Spectrophotometer (*λ *= 260/280 nm; NanoDrop Technologies, Inc., Wilmington, DE). Thereafter, mRNA was converted into cDNA, using a QuantiTect Reverse Transcription Kit (Qiagen, Valencia, CA). Primers were obtained from The DNA Core Facility at Iowa State University (Table 2). An optimal annealing temperature for each primer pair was determined, using a gradient of annealing temperatures in a thermal range of 59–69°C on an Eppendorf Mastercycler RealPlex (Thermo Fischer Scientific, Inc., Waltham, MA). The resulting PCR product was run on a 1% agarose gel to identify the thermal condition resulting in peak expression of a single product. Primer sequences and the corresponding optimized annealing temperatures are shown in Table 2. Relative transcript abundance was assessed using QuantiFast SYBR Green PCR Kit (Qiagen, Valencia, CA) for real‐time qPCR. qPCR plates were placed into an Step One System (Thermo Fischer Scientific, Inc., Waltham, MA) and run at 95°C for 5 min for SYBR activation, 95°C for 10 sec for denaturation followed by annealing and elongation for 30 sec at an optimized annealing temperature (40 cycles). In addition to optimization of run conditions, the quality of the RT‐qPCR was confirmed by assuring a single peak on resultant melting curves. Finally, the product was run on a 1% agarose gel, and a single product of predicted size was obtained. To ensure absence of nonspecific amplification, the product of a no‐template control was separated on a 1% agarose gel, and no bands were detected. Analysis of the CT values was performed, using the ΔΔCT method with 18S as a reference gene.

**Table 2:** Sequences for RT‐qPCR primers

Target	Forward primer	Reverse primer	Ta, °C
18S	ctctagataacctcgggccg	gtcgggagtgggtaatttgc	60.0
IL‐2	ggtgcacctacttcaagctc	ctccctccagagctttgagt	64.4
IL‐6	agatgccaaaggtgatgcca	ctcagggtctggatcagtgc	65.7
IL‐1*β*	ccaaagagggacatggagaa	ttatatcttggcggcctttg	62.2
IL‐8	gaaatcacaggatgcccagt	tgcaagttgaggcaagaaga	61.8
IL‐10	tgtgccctatggtgttcaac	ctttgtcacactccggaagc	59.7
IL‐15	tcctggagttacgcgtcatt	ttttcctccagctcctcaca	62.2
TNF*α*	gcccttccaccaacgttttc	tcccaggtagatgggttcgt	66.9

### Statistical analysis

All data were analyzed, using SAS version 9.2 (SAS Ints. Inc., Cary, NC) of PROC MIXED procedure. The model included time (0, 2, 4, and 6 h of heat stress) as a fixed effect. Additionally, linear and quadratic effects of heat stress were analyzed, using contrast statements of SAS. Data are reported as least square mean ± SEM and considered significant at *P* < 0.05.

## Results

### Phenotypic response

The physiological response to short‐term heat stress in pigs has been previously reported (Pearce et al. 2014). Briefly, animals increased core temperature from 39.2 ± 0.1°C (thermoneutral group) to approximately 41.2 ± 0.1°C at 2 h and remain elevated at 4 h, and 6 h of heat stress (*P* < 0.05). Respiratory rate was increased from 46 ± 2 breaths/minute in the thermoneutral group to 155 ± 44, 151 ± 30, and 135 ± 17 breaths/minute following 2, 4, and 6 h of heat stress, respectively (*P* < 0.05).

### Heat shock proteins

**Figure 1:**
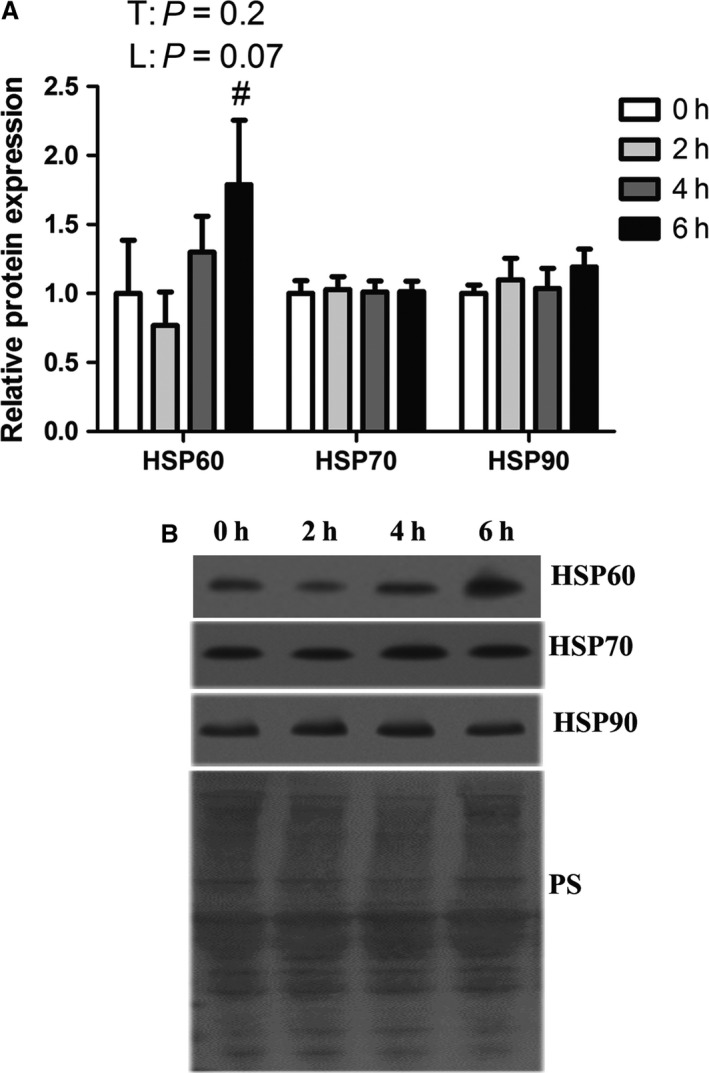
Effect of short‐term heat stress on heat shock proteins in oxidative skeletal muscle. Relative protein abundance of HSP60, HSP70, and HSP90 were measured using western blot (A). Representative blots are included (B). Ponceau S stain (PS) was used as a loading control. Values are mean ± SE;* n* = 8/group. # Indicates significant difference compared to 2 h of heat stress, *P* < 0.05; T indicates time effect between the treatments, L indicates linear effect between the treatments.

To assess the extent of the heat shock protein response to heat stress, we measured relative protein abundance of HSP60, 72, and 90. Both HSP72 and 90 remained similar between groups and HSP60 was increased following 6 h of environmental hyperthermia compared to 2 h (*P* < 0.05). In addition, HSP60 tended to increase linearly with heat stress (*P* = 0.07; Fig. 1A and B).

### Inflammatory signaling

**Figure 2:**
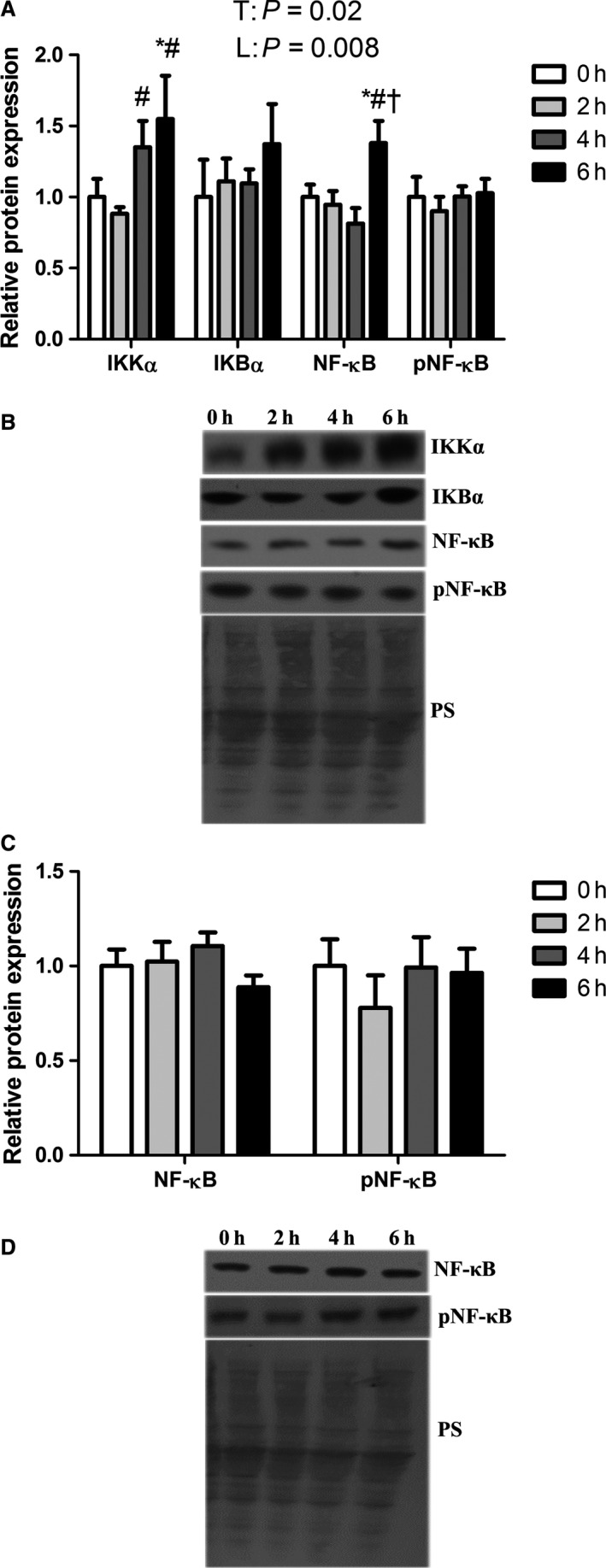
Short‐term heat stress altered NF‐*κ*B pathway in oxidative skeletal muscle. Relative protein abundance of IKK *α*, IKB *α*, NF‐*κ*B and pNF‐*κ*B were measured in whole homogenate (A) and NF‐*κ*B and pNF‐*κ*B were measured in nuclear fraction (C) using western blot. Representative blots are included (B and D). Ponceau S stain (PS) was used as a loading control. Values are mean ± SE;* n* = 8/group. * Indicates significant difference compared to TN control, *P* < 0.05; # Indicates significant difference compared to 2 h of heat stress, *P* < 0.05; † indicates significant difference compared to 4 h of heat stress, *P* < 0.05. T indicates time effect between the treatments, L indicates linear effect between the treatments.

We have previously established increased serum LPS content in these pigs following 2, 4, and 6 h of heat stress (Pearce et al. 2014), which can drive NF‐*κ*B signaling (Guijarro‐Munoz et al. 2014). We discovered that relative protein abundance of the NF‐*κ*B activator, IKK*α*, increased linearly (*P* < 0.05) with progressive hyperthermic exposure to a peak of a 55% increase compared to thermoneutral animals (Fig. 2A and B). IKK*α* phosphorylates IKB*α*, an endogenous NF‐*κ*B inhibitor, and leads to its subsequent degradation. Protein abundance of IKB*α* was similar between groups. Total NF‐*κ*B protein abundance was increased by approximately 40% following 6 h of heat stress compared to all other groups, which were similar to each other (*P* < 0.05; Fig. 2A and B). Protein abundance of phosphorylated NF‐*κ*B was similar between groups. Relative abundance of total and phosphorylated NF‐*κ*B in the nuclear fraction was also similar between groups (Fig. 2C and D).

**Figure 3:**
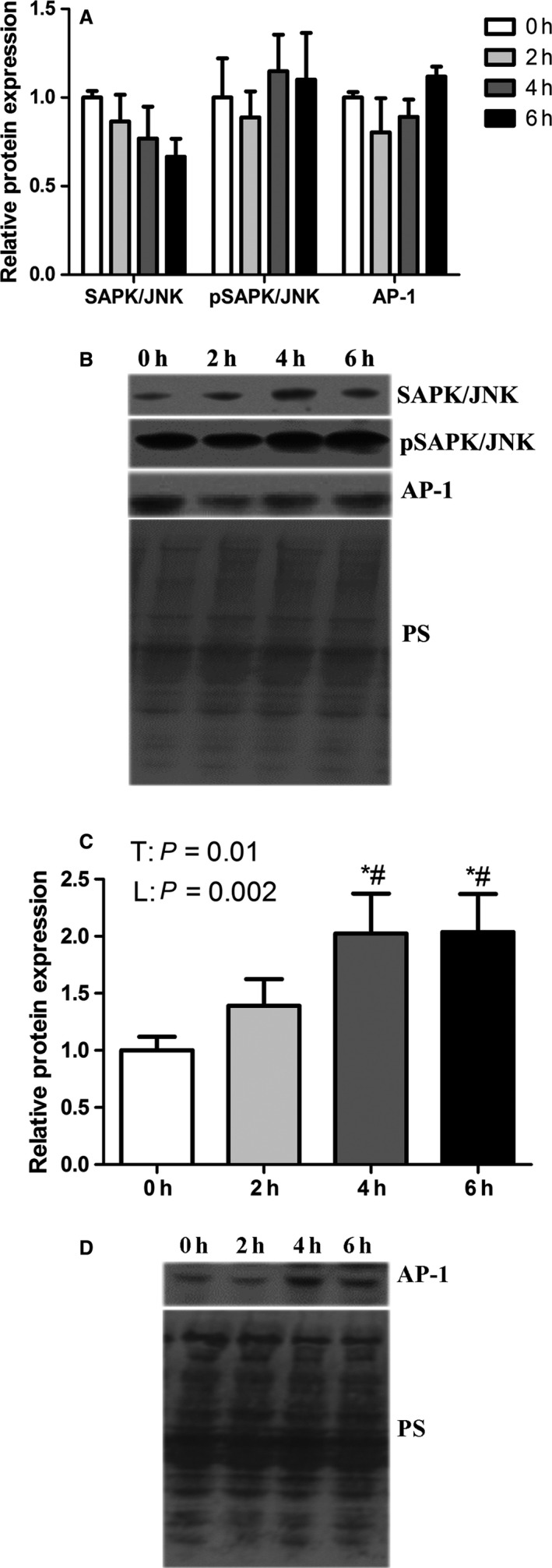
Short‐term heat stress altered AP‐1 signaling in oxidative skeletal muscle. Relative protein abundance of SAPK/JNK, pSAPK/JNK and AP‐1 were measured in whole homogenate (A) and AP‐1 was measured in nuclear fraction (C) using western blot. Representative blots are included (B and D). Ponceau S stain (PS) was used as a loading control. * Indicates significant difference compared to TN control, *P* < 0.05. # Indicates significant difference compared to 2 h of heat stress, *P* < 0.05; T indicates time effect between the treatments, L indicates linear effect between the treatments Values are mean ± SE;* n* = 8/group.

To determine the extent to which environmental hyperthermia induced AP‐1 signaling we assessed activation and localization of pathway components. Relative protein abundance of SAPK/JNK and phosphorylated SAPK/JNK kinase were similar between groups as was AP‐1 (Fig. 3A and B). Interestingly, we found that nuclear AP‐1 relative protein abundance increased linearly (*P* < 0.05) such that following 6 h of hyperthermic exposure, nuclear AP‐1 protein abundance was double that of the thermoneutral group (Fig. 3C and D).

**Figure 4:**
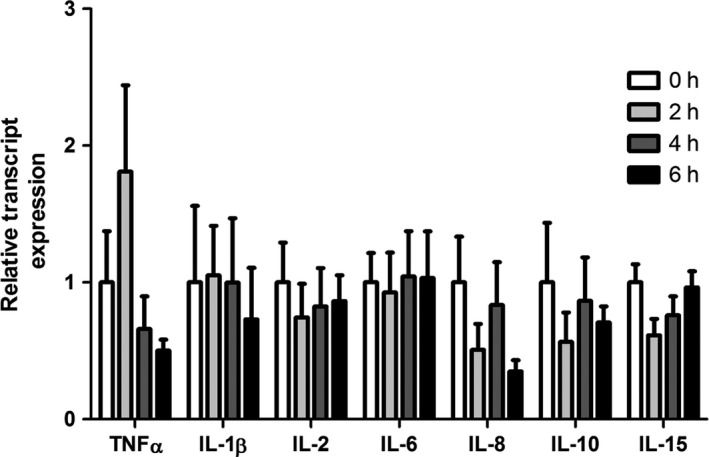
Short‐term heat stress did not change transcript expression of NF‐*κ*B‐ and AP‐1‐driven proinflammatory cytokines in oxidative skeletal muscle. Relative transcript expression was measured for proinflammatory cytokines using qRT‐PCR in oxidative muscle. Values are fold change ±SE;* n* = 8/group.

**Figure 5:**
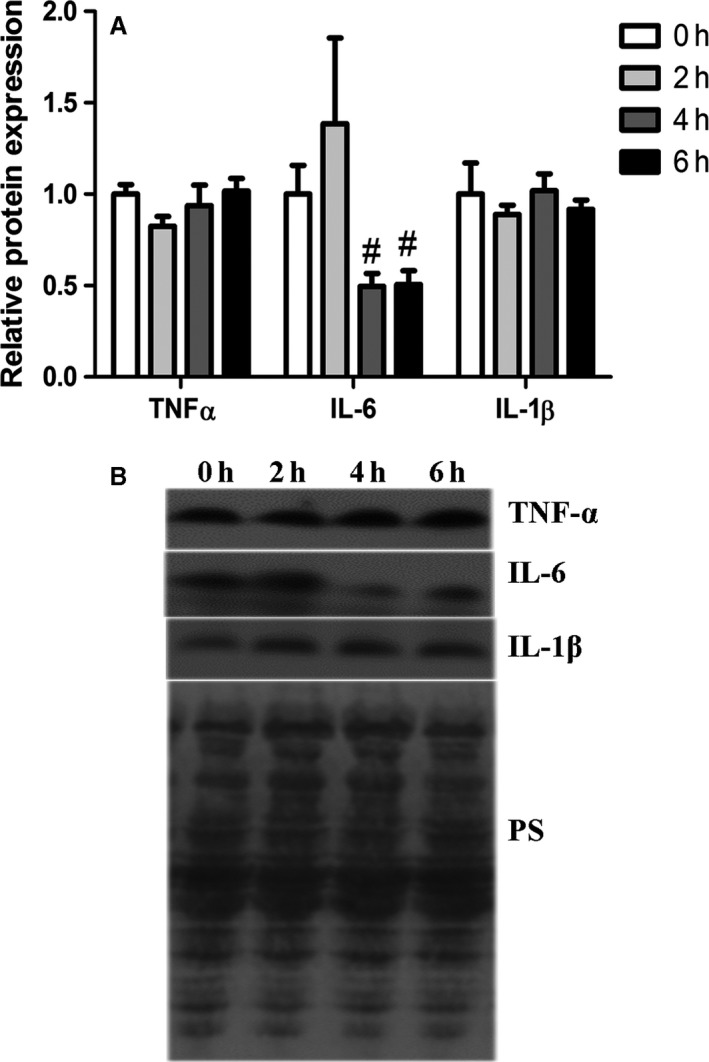
Short‐term heat stress altered relative protein expression of IL‐6 in oxidative porcine skeletal muscle. Relative protein abundance of TNF *α*, IL‐1*β* and IL‐6 were measured using western blot (A). Representative blots are included (B). Ponceau S stain (PS) was used as a loading control. Values are mean ± SE;* n* = 8/group. # Indicates significant difference compared to 2 h heat stress, *P* < 0.05

The end product of NF‐*κ*B and AP‐1 signaling is increased abundance of a variety of transcripts and resultant proteins, including cytokines. Relative transcript expression of select cytokines driven by NF‐*κ*B and/or AP‐1 was similar between groups (Fig. 4). Relative protein abundance of TNF*α* and IL‐1*β* was similar between groups (Fig. 5A and B). However, relative protein abundance of IL‐6 was decreased by 49% and 48%, respectively, following 4 and 6 h of heat stress compared to 2 h of heat stress (*P* < 0.05; Fig. 5A and B).

**Figure 6:**
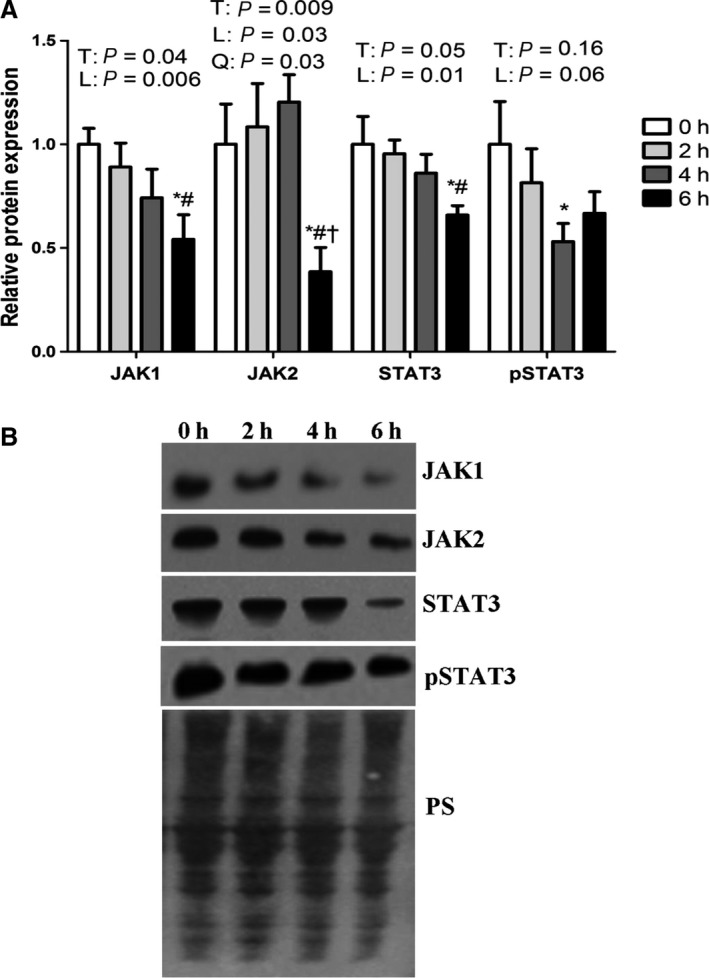
Short‐term heat stress altered JAK/STAT relative protein expression in oxidative porcine skeletal muscle. Relative protein abundance of JAK1, JAK2, STAT3 and pSTAT3 were measured using western blot (A). Representative blots are included (B). Ponceau S stain (PS) was used as a loading control. Values are mean ± SE;* n* = 8/group. * indicates significant difference compared to TN control, *P* < 0.05. # Indicates significant difference compared to 2 h of heat stress, *P* < 0.05; † Indicates significant difference compared to 4 h of heat stress, *P* < 0.05. T indicates time effect between the treatments, L indicates linear effect between the treatments, Q indicates quadratic effect between the treatment.

Cellular effects of IL‐6 involve activation of the Janus kinases (JAKs) and transcription factors of the STAT family (Bellido et al. 1997; Heinrich et al. 1998). We found that 6 h of heat stress linearly decreased JAK1 protein abundance (*P* < 0.05) reaching a nadir of 46% of thermoneutral following 6 h of exposure (Fig. 6A and B). Protein abundance of JAK2 had a quadratic relationship (*P* < 0.05) such that it increased linearly (*P* < 0.05) following 2 and 4 h of heat stress, however, fell to a nadir of 38% of thermoneutral following 6 h of heat stress (Fig. 6A and B). STAT3 protein abundance was decreased linearly (*P* < 0.05) throughout the environmental treatment and by 6 h fell by 35% compared to thermoneutral (Fig. 6A and B). Consistent with the notion of decreased IL‐6 protein abundance, phosphorylated STAT3 protein abundance was decreased by 34% following 4 h of environmental hyperthermia compared to thermoneutral group and tended to decrease linearly throughout the 6 h intervention (*P* = 0.06).

## Discussion

Prolonged exposure to elevated environmental temperature causes heat stress in humans and animals, which compromises health (Kones 2011) and negatively impacts animal welfare (St‐Pierre et al. 2003; Pearce et al. 2013c, 2014) and agricultural economics (St‐Pierre et al. 2003; Key et al. 2014). Aside from prevention, cooling and rehydration are used to treat heat stress‐induced injury (Miners 2010). Etiological treatment is currently unavailable, in part, because of limited knowledge regarding intracellular mechanisms of heat related injury. In oxidative skeletal muscle we have previously shown a transition from activated NF‐*κ*B signaling following 12 h of heat stress (Ganesan et al. 2016) to quiescence following 24 h of heat stress (Montilla et al. 2014). AP‐1 signaling did not appear to be active following 12 h of heat stress, however, we discovered evidence of prior pathway activity (Ganesan et al. 2016). These findings suggested that duration of environmental hyperthermia impacts inflammatory signaling in skeletal muscle. Here, we addressed the hypothesis that 2, 4, and 6 h of heat stress would lead to inflammatory signaling via the NF‐*κ*B and AP‐1 pathways in oxidative skeletal muscle. In partial support of our hypothesis we found early signs of NF‐*κ*B pathway activation as well as increased AP‐1 signaling.

Previously, we showed that 12 h of heat stress was characterized by increased NF‐*κ*B pathway activity including stimulated NF‐*κ*B protein abundance in the nuclear fraction and increased expression of NF‐*κ*B‐driven genes (Ganesan et al. 2016). In total, our discoveries in this investigation, combined with our previous observations, provide a picture of gradual heat stress‐mediated activation of NF‐*κ*B signaling. Of note, between 6–12 h of heat stress pathway activation ostensibly returns to thermoneutral conditions, however, NF‐*κ*B already present in the nuclei continues to drive NF‐*κ*B‐responsive genes. We previously established that 24 h of heat stress no longer activated NF‐*κ*B signaling (Montilla et al. 2014), suggesting that between 12–24 h inhibition/deactivation of these NF‐*κ*B‐sensitive genes occurs. Such NF‐*κ*B signaling during prolonged environmental hyperthermia is in stark contrast to events occurring during more acute exposures with heat stroke. For example, 60 min of heat exposure upregulated IKB*α*, a suppressor of NF‐*κ*B (Ohno et al. 2010), and in muscle NF‐*κ*B signaling did not appear to contribute to dysfunction in heat stroke models (Welc et al. 2012, 2013b).

Inflammatory signaling may also be driven by AP‐1 (Diamond et al. 1999; Welc et al. 2013a). In a heat stroke model, 1 h of hyperthermic exposure was sufficient to induce activation in SAPK signaling in skeletal muscle (Welc et al. 2013b) and AP‐1 protein abundance was increased by 15 (Diamond et al. 1999) and 30 min (Welc et al. 2013a) of hyperthermia. In our current study, we found evidence supporting activated AP‐1 signaling as relative protein abundance of AP‐1 was increased in nuclear fractions following 4 and 6 h of heat stress compared to thermoneutral. These events suggest that following 4 h of heat stress, SAPK/JNK promotes translocation of the AP‐1 protein into the nuclei. Of interest, we reported previously that following 12 h of heat stress, AP‐1 signaling was inactive in oxidative muscle (Ganesan et al. 2016), however, expression of AP‐1‐driven transcripts continued to be increased. We proposed that increased transcript expression was likely due to previous activation of the AP‐1 pathway, which is now supported given these present findings. This also suggests that between 6 and 12 h of heat stress, intracellular inflammatory signaling via AP‐1 shifts from an active to inactive state.

In addition to AP‐1 and NF‐*κ*B signaling, changes in IL‐6 transcript and protein abundance may represent a key difference between acute (heat stroke) and prolonged exposure to environmental hyperthermia. In several studies modeling heat stroke IL‐6 has been shown to be increased and cytoprotective (Leon 2007; Welc et al. 2012, 2013b; Phillips et al. 2015). In contrast, during 4 and 6 h and more prolonged hyperthermic exposures (Montilla et al. 2014; Ganesan et al. 2016), IL‐6 protein abundance was decreased potentially contributing to multisystem dysfunction. Moreover in the present investigation, heat stress‐mediated reductions in IL‐6 protein abundance were closely mirrored by reductions in JAK1, JAK2, STAT3, and p‐STAT3 protein abundance. IL‐6 can regulate JAK/STAT signaling (Bellido et al. 1997; Heinrich et al. 1998) and consistent with our data, a hyperthermic suppression of JAK/STAT signaling has been previously reported (Nespital and Strous 2012).

TNF*α* activates the NF‐*κ*B pathway and is also produced as a result of its activity (Ghosh et al. 1998). We reported previously that 12 to 72 h of heat stress increased TNF*α* 2 to 2.5‐fold in skeletal muscle (Montilla et al. 2014; Ganesan et al. 2016). We speculated this increase resulted from early changes in inflammatory signaling or migration from vasculature since TNF*α* blood content decreased following 12 and 24 h of heat stress (Pearce et al. 2013b, 2015) while muscle transcript expression was not elevated (Montilla et al. 2014; Ganesan et al. 2016). In the current experiment, 6 h of heat stress was insufficient to increase TNF*α* protein or transcript abundance, but this timeframe was characterized by activated NF‐*κ*B signaling. Despite apparent changes in circulating TNF*α* in the same pigs (Pearce et al. 2014), skeletal muscle TNF*α* abundance following 6 h of heat stress is ostensibly independent of NF‐*κ*B signaling.

It is important to consider these changes within the context of what has been previously discovered about these animals. Germane to this investigation, heat stress resulted in a progressive increase in endotoxemia concomitant with intestinal damage and leakage (Pearce et al. 2014). While it is likely that endotoxemia contributed to inflammatory signaling in these muscles the possibility of other mediators cannot be eliminated. For example, we also found increased oxidative stress in these tissues (Volodina et al. 2017) and oxidative stress has been previously shown to lead to inflammatory signaling (Pizza et al. 1998). In addition, in skeletal muscle heat stress failed to increase expression of heat shock proteins, however in intestinal samples from these animals they were elevated (Pearce et al. 2014). It is unclear what underlies the differing heat shock protein response though speculatively, in addition to elevated temperatures, the gut may also experience ischemic injury (Pearce et al. 2014) while muscle may not. In addition, our heating intervention may not increase muscle temperatures beyond some threshold experienced during contraction and therefore may not represent a thermal stress sufficient to induce expression of heat shock proteins despite elevated core temperatures.

In summary, short‐term heat stress activated inflammatory signaling in the STR chronologically such that AP‐1 was activated prior to NF‐*κ*B. In contrast to heat stroke models prolonged hyperthermic exposure decreased IL‐6 protein abundance, which may blunt JAK/STAT signaling. From a practical perspective, these data indicate that hyperthermic muscle injury is associated with inflammatory signaling, which may serve as an initiating event for subsequent changes at later time points. While targeting inflammatory signaling may be a simple solution, it may also limit production of IL‐6, which appears to provide systemic protection during environmental hyperthermia, at least under heat stroke conditions, and depending on anti‐inflammatory agent, may also exacerbate intestinal injury. Nevertheless, given the broad, negative impacts of hyperthermia to humans and animals alike, and the widespread availability of anti‐inflammatories, empirical testing of this hypothesis should be considered.

## Conflict of Interest

The authors have no conflicts to declare.
